# Discovering new genes for alfalfa (*Medicago sativa*) growth and biomass resilience in combined salinity and *Phoma medicaginis* infection through GWAS

**DOI:** 10.3389/fpls.2024.1348168

**Published:** 2024-05-02

**Authors:** Wiem Mnafgui, Cheima Jabri, Nada Jihnaoui, Nourhene Maiza, Amal Guerchi, Nawres Zaidi, Gerhard Basson, Eden Maré Keyster, Naceur Djébali, Luciano Pecetti, Mohsen Hanana, Paolo Annicchiarico, Muhammet Sakiroglu, Ndiko Ludidi, Mounawer Badri

**Affiliations:** ^1^ Laboratory of Extremophile Plants, Centre of Biotechnology of Borj Cedria, Hammam-Lif, Tunisia; ^2^ Faculty of Sciences of Tunis, University of Tunis El Manar, El Manar Tunis, Tunisia; ^3^ Environmental Biotechnology Laboratory, Department of Biotechnology, University of the Western Cape, Bellville, South Africa; ^4^ Plant Stress Tolerance Laboratory, University of Mpumalanga, Mbombela, South Africa; ^5^ Laboratory of Bioactive Substances, Centre of Biotechnology of Borj Cedria, Hammam-Lif, Tunisia; ^6^ Council for Agricultural Research and Economics, Research Centre for Animal Production and Aquaculture, Lodi, Italy; ^7^ Department of Bioengineering, Adana Alparslan Türkeş Science and Technology University, Adana, Türkiye; ^8^ DSI-NRF Centre of Excellence in Food Security, University of the Western Cape, Bellville, South Africa

**Keywords:** alfalfa, biomass traits, genetic resources, GWAS, *Phoma medicaginis*, salinity, stress tolerance

## Abstract

Salinity and *Phoma medicaginis* infection represent significant challenges for alfalfa cultivation in South Africa, Europe, Australia, and, particularly, Tunisia. These constraints have a severe impact on both yield and quality. The primary aim of this study was to establish the genetic basis of traits associated with biomass and growth of 129 *Medicago sativa* genotypes through genome-wide association studies (GWAS) under combined salt and *P. medicaginis* infection stresses. The results of the analysis of variance (ANOVA) indicated that the variation in these traits could be primarily attributed to genotype effects. Among the test genotypes, the length of the main stem, the number of ramifications, the number of chlorotic leaves, and the aerial fresh weight exhibited the most significant variation. The broad-sense heritability (*H*²) was relatively high for most of the assessed traits, primarily due to genetic factors. Cluster analysis, applied to morpho-physiological traits under the combined stresses, revealed three major groups of accessions. Subsequently, a GWAS analysis was conducted to validate significant associations between 54,866 SNP-filtered single-nucleotide polymorphisms (SNPs) and seven traits. The study identified 27 SNPs that were significantly associated with the following traits: number of healthy leaves (two SNPs), number of chlorotic leaves (five SNPs), number of infected necrotic leaves (three SNPs), aerial fresh weight (six SNPs), aerial dry weight (nine SNPs), number of ramifications (one SNP), and length of the main stem (one SNP). Some of these markers are related to the ionic transporters, cell membrane rigidity (related to salinity tolerance), and the NBS_LRR gene family (associated with disease resistance). These findings underscore the potential for selecting alfalfa genotypes with tolerance to the combined constraints of salinity and *P. medicaginis* infection.

## 1 Introduction

Tunisia boasts a rich and diverse range of forage and pasture biodiversity, with over 960 pasture legume species, including 336 that are unique to the Mediterranean region (Ibidhi et al., 2018; Ferchichi et al., 2021). However, due to increasing purchasing power, the demand for ruminant products is expected to rise significantly in the coming years, making high levels of forage crop production imperative. The country’s livestock sector heavily relies on forage crops, particularly alfalfa (*Medicago sativa*), which covers an area of approximately 12,410 hectares (Guiza et al., 2022). Alfalfa plays a vital role in supporting livestock nutrition and ensuring food security.

Presently, the Tunisian livestock sector is grappling with a persistent shortage of forage. Like many other regions globally, it faces the challenge of climate change and its associated environmental stresses, among which soil salinization and pathogen infections are particularly important for alfalfa productivity (Djebali, 2008; Arraouadi et al., 2011; Badri et al., 2021). Furthermore, the interaction between salinity and *Phoma medicaginis* infection can exacerbate the adverse effects of these stresses on alfalfa plants (Castell-Miller et al., 2007; Kaiwen et al., 2020; Maiza et al., 2021). The coexistence of these stresses creates a more hostile environment for the crop to thrive. Under saline conditions, reduced water uptake and compromised nutrient absorption weaken the plant’s defenses against strain *Phoma medicaginis* 8 (Pm8) infection, making the plant more susceptible to disease progression (Djébali et al., 2013). Therefore, the increasing prevalence of salinity stress and *P. medicaginis* infection poses a significant threat to alfalfa production in Tunisia.

Despite its significance as a forage legume, there has been limited work on varietal selection for this species in Tunisia, with only two registered local varieties (Gabès and El Hamma) (Jabri et al., 2021; Maiza et al., 2021). Therefore, it is advisable to initiate new varietal selection efforts for alfalfa, utilizing diverse germplasm, to develop new varieties with robust agronomic performance under the combined constraints of salinity and *P. medicaginis* infection in the context of climate change.

Moreover, the socio-economic implications of the shortage of forage in Tunisia, particularly its impact on the livestock sector and food security, warrant further exploration and discussion.

As a result, diverse studies focusing on enhancing alfalfa have led to the development of cultivars showcasing superior attributes such as increased yield, enhanced stress tolerance, and improved forage quality (Badri et al., 2021). A significant challenge in alfalfa enhancement lies in pinpointing the genetic underpinnings responsible for variations within the species across various agronomic traits (Sakiroglu and Brummer, 2017). Addressing these implications is essential for devising effective strategies to mitigate the challenges faced by farmers and ensure sustainable agricultural practices.

Furthermore, a comparative analysis of alfalfa production and management strategies such as cropping systems-, and soil-related management practices (Thivierge et al., 2023) in other regions facing similar environmental stresses could provide valuable insights and lessons learned that can inform the development of tailored solutions for Tunisia and similar ecogeographic regions (Tlahig et al., 2020). By examining approaches adopted in different contexts, we can identify best practices and adapt them to the Tunisian agricultural landscape, thereby enhancing the resilience and productivity of the alfalfa sector.

Alfalfa is an autotetraploid species with a chromosome count of 32 (2n = 4x = 32) and a genome size ranging from 800 to 1000 Mb. Several challenges affect its genetic improvement because of various factors such as intra-varietal variation, environmental influences, and the complex life cycle of the plant (Liu et al., 2023). Due to these hindrances, conventional breeding in alfalfa has been difficult and only moderately efficient (Annicchiarico et al., 2015a). The progression of genomic technology could play a pivotal role in allowing association mapping studies that could accelerate and strengthen crop improvement (Zhang et al., 2020).

Genomic investigations, including those based on genotyping-by-sequencing (GBS) markers, have enabled the detection of QTLs that influence various important agricultural characteristics in alfalfa. These traits encompass biomass yield, tolerance to factors such as aluminum, drought, and salt, as well as the ability to withstand freezing and maintain biomass yield during drought stress (Filho et al., 2023). Genome-wide association studies (GWAS) offer a potent strategy for deciphering genomic regions involved in targeted traits by scrutinizing the association between genome-wide single-nucleotide polymorphism (SNP) markers and phenotypic variation with exceptional precision compared to other approaches (Porter and O’Reilly, 2017; Larkin et al., 2019; Lin et al., 2023). Indeed, the GWAS approach enables the detection of more than one distinct allele for each locus in the studied accessions, and it would allow for a much more precise identification of candidate genes potentially involved in the genetic determination of the agronomic traits of interest (Liu et al., 2023).

Previously, GWAS has been employed in alfalfa to pinpoint loci markers associated with forage yield (Sakiroglu et al., 2012), drought resistance (Zhang et al., 2015), salt tolerance (Yu et al., 2016), forage quality (Sakiroglu and Brummer, 2017; Biazzi et al., 2017), and resistance to *Verticillium* wilt (Yu et al., 2017). No information is available for candidate genes underlying possible alfalfa tolerance to combined stresses of *P. medicaginis* infection and salinity.

The current work aimed to (i) characterize the response of alfalfa accessions to the combination of salinity and *P. medicaginis* infection, and (ii) identify the genetic determinants of tolerance to these constraints in this species through GWAS, to predict breeding values using SNP markers with allele dosage in breeding populations of alfalfa.

## 2 Materials and methods

### 2.1 Plant materials and phenotyping

A set of 126 alfalfa parent plants was randomly chosen from a Mediterranean reference population obtained from crosses between the elite cultivars (Erfoud 1, Mamuntanas, and Sardi 10) over two generations. These cultivars have demonstrated strong adaptation and different adaptive patterns in Mediterranean environments (Annicchiarico et al., 2011). These plants underwent *Apek*I-based GBS characterization as described in Annicchiarico et al. (2015b), and were polycrossed in isolation to obtain half-sib progeny seed used for phenotyping experiments. The seeds of these half-sib families (named as “accessions” hereafter) and those of the three parent cultivars were germinated in Petri dishes within a culture chamber for six days. After the radicle appearance, they were transplanted in 1-liter pots (diameter of 22.8 cm and height of 18 cm) filled with a mixture of soil and compost (2: 1) in a greenhouse with a temperature of 25/18°C (day/night), a relative humidity of 60–80%, and a photoperiod of 16/8 hours (light/dark). After 2 months of cultivation, plants at the flowering bud stage were subjected to a saline treatment of 150 mM NaCl (for 21 days) until the appearance of symptoms related to saline stress. At this stage, they were infected with the strain Pm8 of *Phoma medicaginis* (5*10^6^ conidies µL^-1^) as described by Zaidi et al. (2021). Previous optimization studies by Badri et al. (2021) and Jabri et al. (2021) on alfalfa grown under a range of salt concentrations (NaCl) have revealed that 150 mM NaCl is the most appropriate for analyzing the behavior of accessions of this species under this constraint. The rationale for starting the inoculation 21 days after the salt treatment is based on previous research findings (Haddoudi et al., 2021) that indicate a specific timeframe during which the plants exhibit maximum susceptibility to infection post-salt stress exposure. This interval ensures that the plants are at a stage where their physiological responses to salt stress have been initiated, thereby facilitating a more accurate assessment of the interaction between salt stress and pathogen infection. The trial was conducted with a split-plot design, employing a completely randomized setup with three distinct blocks. Within each block, three replicates were allocated for every genotype (129 genotypes), and the plants were cultivated in pots within a greenhouse environment. This resulted in a total of 1,161 plants, with nine replicates for each accession and treatment.

Fourteen days after the disease inoculation, all the accessions were screened for a set of seven morphological traits, including four traits related to growth and biomass parameters (aerial fresh weight, AFW; aerial dry weight, ADW; length of the main stem, LMS; and number of ramifications, NR) and three traits related to the leaf aspect (number of chlorotic leaves, NCL; number of healthy leaves, NHL; and number of chlorotic necrotic leaves, NCNL).

### 2.2 Phenotypic data analyses

An ANOVA was performed for each trait to assess the response of accessions to combined salt and strain Pm8 infection stresses, testing the equality of variance and marginal means. Meanwhile, a normality test was conducted for each trait using the Kolmogorov-Smirnov tests. Pearson correlation was calculated between traits using trait means. All analyses were performed using SPSS 20 (http://www.ibm.com/software/analytics/spss/). Broad-sense heritability (*H* ^2^) was estimated using the formula:


$$H^{2}=Vg/\left(Vg+Ve\right)$$

where Vg and Ve were genotypic variance and environmental variance, respectively.

The genotypic coefficient of variation (GCV) was calculated using the formula:


 GCV(%)=VgX*100

where X is the average of each trait of the whole association panel (Ahsan et al., 2015).

Furthermore, a principal component analysis (PCA) and a hierarchical clustering analysis (HCA) were performed to identify the grouping of the measured traits and the patterns of differentiation among accessions under combined stresses. The PCA and the HCA were performed using XLSTAT version 2022 based on the standardized mean values of all traits.

### 2.3 Alignment to *Medicago truncatula*, GWAS and marker-trait association analyses

We estimated suitable LS-means values and applied them to standardize our phenotypic data, ensuring that the prerequisite of normality for conducting genome-wide association analyses was met. Following this standardization process, we conducted the Kolmogorov-Smirnov test for each phenotype to confirm the attainment of a normal distribution in our phenotypic data.

With no alfalfa reference currently available, the *Medicago truncatula* genome may be a potential reference genome, owing to the presence of a high level of synteny between the *M. sativa* linkage map and the physical map of *M. truncatula* due to its small genome size (Li et al., 2015). SNP markers identified by GBS underwent an additional filtration step, wherein those with a minor allele frequency (MAF) below 0.05 were excluded. The resulting set of 54,866 SNPs was employed to conduct an analysis of associations between markers and traits using the TASSEL software v5.0 (http://www.maizegenetics.net/tassel) (Bradbury et al., 2007) for the GWAS. To account for potential population structure effects, a mixed linear model (MLM) incorporating a kinship (K) matrix was employed during the association mapping process. Statistical significance of markers was ascertained using the false discovery rate (FDR) method with a threshold of 0.05, as outlined by Benjamini and Hochberg (1995).

False discovery rate in GWAS may be controlled by Bonferroni’s multiple testing correction method, which proved to be too conservative in practice. We preferred to use a conservative P level corresponding to an association score [–Log10 (p-value)] 4, as suggested in earlier studies (Kang et al., 2015; Sakiroglu and Brummer, 2017). *M. truncatula*-aligned SNPs that were significantly associated with one or more alfalfa studied traits were mapped on Mt4.0v1 using the Jbrowse tool in the plant comparative genomics portal ensemble Plants (http://plants.ensembl.org/index.html) to identify putative genes with significant SNPs, searching through 10 kb upstream and downstream of each SNP. Knowledge on the function of those genes is useful for selecting significant SNPs to integrate into breeding programs for targeted traits.

## 3 Results

### 3.1 Morphophysiological variation, heritability, and correlations among traits

Results from ANOVA showed a significant effect of the genotype factor on all measured traits (**Table 1**). Notably, this factor exerted a strong influence on both the morphological parameters related to growth and biomass production (LMS, NR, AFW, and ADW) and the leaf traits affected by the combination of Pm8 inoculation and salt stress (NHL, NCL, NCNL). A moderate to high coefficient of variation (CV) was noted among genotypes for LMS (22.01%), ADW (55.43%), NCL (52.87%) and NHL (50.48%). Overall, the broad-sense heritability (*H* ^2^) was relatively high for most traits under combined stresses. It ranged from 0.35 for NCL to 0.84 for NHL, with all other traits showing an intermediate heritability value of about 0.5-0.6 (**Table 2**).

**Table 1:** Influence of accession, block, and the interaction between accession and block on the variability of morphological traits measured for alfalfa (*Medicago sativa*) accessions cultivated under the combination of salinity and *Phoma medicaginis* (strain Pm8) infection.

			Genotype		Block		Genotype x Block		
Traits	Mean	DF	F	*P*	F	*P*	F	*P*	CV
**LMS**	47.27	128	2.52	<0.0001	3.19	0.041	1.71	0.000	22.01
**NR**	8.093	128	2.35	<0.0001	43.49	<0.0001	2.11	<0.0001	37.72
**NHL**	16.64	128	1.54	0.001	4.44	0.012	1.21	0.098	50.48
**NCL**	17.94	128	1.53	0.001	25.84	<0.0001	1.03	0.405	52.78
**NCNL**	16.49	128	2.05	<0.0001	16.75	<0.0001	1.62	0.001	45.31
**AFW**	7.45	128	2.31	<0.0001	14.37	<0.0001	1.83	<0.0001	51.53
**ADW**	2.29	128	2.03	<0.0001	6.29	0.002	2.3	<0.0001	55.43

F is the coefficient of Snedecor-Fisher with significance at P ≤ 0.05. Length of medium stem (LMS, cm), number of ramification (NR), number of healthy leaves (NHL), number of chlorotic leaves (NCL), number of chlorotic necrotic leaves (NCNL), aerial fresh weight (AFW, g), and aerial dry weight (ADW, g). CV, Coefficient of variation

**Table 2:** Genotypic variation (Vg), environmental variation (Ve), and heritability (*H* ^2^) of seven morphological traits in alfalfa (*Medicago sativa*) accessions cultivated under the combination of salinity and *P. medicaginis* (strain Pm8) infection.

Trait	Vg	Ve	*H* ^2^
**LMS**	15.69	92.59	0.61
**NR**	1.22	8.11	0.57
**NHL**	0.54	0.93	0.84
**NCL**	4.98	84.48	0.35
**NCNL**	5.83	49.99	0.51
**AFW**	1.88	12.9	0.56
**ADW**	0.16	1.45	0.51

Length of medium stem (LMS, cm), number of ramification (NR), number of healthy leaves (NHL), number of chlorotic leaves (NCL), number of chlorotic necrotic leaves (NCNL), aerial fresh weight (AFW, g), and aerial dry weight (ADW, g).

Of the 21 possible pairwise correlations, 20 were significant and positive (**Table 3**). Noticeably, a significant positive correlation was also observed between NHL and NCNL (r=0.668; *P ≤* 0.0001) which seems to indicate that NCNL is not an independent parameter due to the effect of combined stresses but it is simply related to the potential number of healthy leaves that the genotype is able to express.

**Table 3:** Correlation matrix of seven morphological traits for alfalfa (*Medicago sativa*) accessions cultivated under the combination of salinity and *Phoma medicaginis* (strain Pm8) infection.

	LMS	NR	NHL	NCL	NCNL	AFW	ADW
**LMS**	1.000						
**NR**	0.038	1.000					
**NHL**	0.364**	0.336**	1.000				
**NCL**	0.323**	0.303**	0.612**	1.000			
**NCNL**	0.415**	0.350**	0.668**	0.576**	1.000		
**AFW**	0.465**	0.393**	0.757**	0.623**	0.827**	1.000	
**ADW**	0.535**	0.349**	0.693**	0.569**	0.772**	0.879**	1.000

**Significant at P ≤ 0.01. Length of medium stem (LMS, cm), number of ramification (NR), number of healthy leaves (NHL), number of chlorotic leaves (NCL), number of chlorotic necrotic leaves (NCNL), aerial fresh weight (AFW, g), and aerial dry weight (ADW, g).

### 3.2 Principal component analysis and cluster analysis

**Figure 1:**
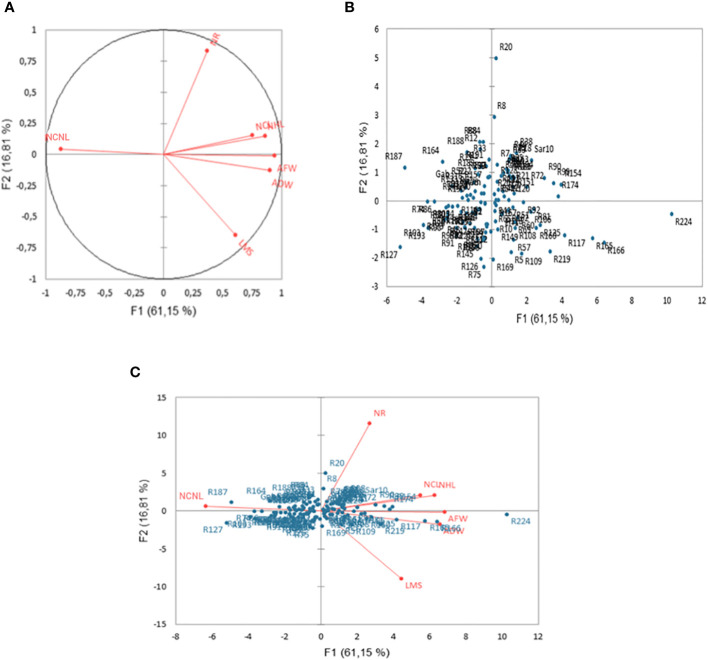
Two-dimensional PCA plot showing the relationships among measured traits **(A)** for the accessions **(B)** and Biplot (genotype, trait) **(C)** representing alfalfa (*Medicago sativa*) under a combination of salinity and *Phoma medicaginis* (Strain Pm8) infection. Length of medium stem (LMS, cm), number of ramifications (NR), number of healthy leaves (NHL), number of chlorotic leaves (NCL), number of chlorotic necrotic leaves (NCNL), aerial fresh weight (AFW, g), and aerial dry weight (ADW, g).

PCA explained most of the variation among genotypes by the first two axes (PC1 and PC2), which represented 61.15% and 16.81%, of the total variance, respectively (**Figure 1**). All the recorded characters were positively correlated with PC1 (r > 0.54) except NCNL, which displayed a high negative association with this axis (**Figure 1A**). Surprisingly, NCNL had a contrasting bearing on PC1 compared with the two other leaf-related traits (NHL and NCL) despite the positive phenotypic correlations among the three leaf traits (**Table 2**). PC1 was strictly associated with the two traits related to biomass production (AFW and ADW), whereas the second axis was somewhat associated to the traits related to plant morphology (NR) (**Figure 1A**). The genotypes were found mostly in an extensive overlap around the origin in both sides of PC1 and PC2 with a few scattered genotypes. The two outlier genotypes R20 and R224, in particular, showed peculiar traits that contributed to their position on the PC plan. R20 had the highest number of ramification and R224 the maximum AFW, ADW, LMS, and the minimum NCL followed by R166, R165 and R174 (**Figure 1B**). The rest of the genotypes appeared not clearly separated from each other on the basis of the measured traits and were more or less equally partitioned into the four quadrants of the PCA (**Figures 1B, C**).

### 3.3 GWAS and marker-trait association analyses

**Figure 2:**
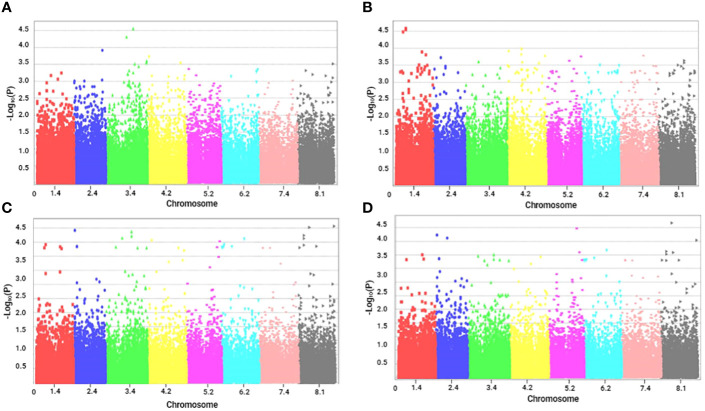
Manhattan plots of marker–trait associations for tolerance traits of alfalfa (*M. sativa*) to a combination of salinity and *Phoma medicaginis* (strain Pm8) infection. Significant markers that passed a cutoff log (p-value) of 4. The X-axis represents the physical location of SNPs on chromosomes (color-coded) and the Y-axis represents the −log10 p-value of SNP phenotype associations. **(A)** length of the main stem (LMS, cm); **(B)** number of ramifications (NR), **(C)** aerial fresh weight (AFW, g) and **(D)** aerial dry weight (ADW, g).

**Figure 3:**
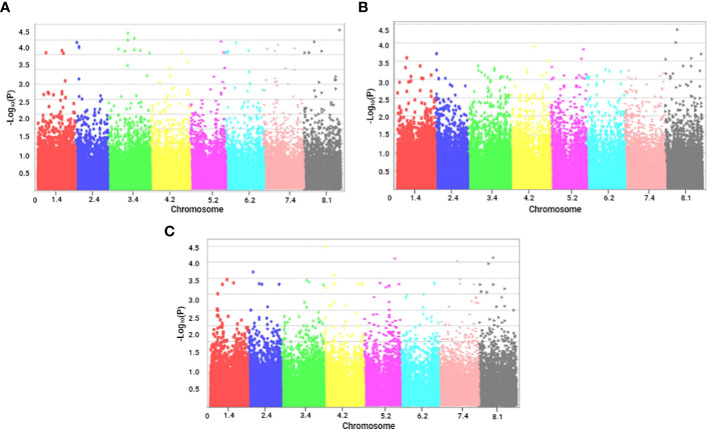
Manhattan plots of marker–trait associations for tolerance traits of alfalfa (*M. sativa*) to a combination of salinity and *Phoma medicaginis* (Strain Pm8) infection. Significant markers that passed a cutoff log (p-value) of 4. The X-axis represents the physical location of SNPs on chromosomes (color-coded) and the Y-axis represents the −log10 p-value of SNP phenotype associations. **(A)** number of chlorotic leaves (NCL); **(B)** number of chlorotic necrotic leaves (NCNL); **(C)** number of healthy leaves (NHL).

The Manhattan plots (**Figures 2**, **3**) illustrate the p-values (transformed to negative logarithmic values) of markers in relation to their genetic positions for each trait. We identified 110 significant SNPs across the seven recorded traits. These SNPs were distributed across all chromosomes, with the maximum of 24 SNPs that were located on chromosome 3 and the minimum of 10 markers on chromosome 2.

Among the growth and biomass traits, the characters LMS (**Figure 2A**) and NR (**Figure 2B**) had two significant markers each, distributed on chromosome 1 and 3, respectively. The highest number of significant markers (35) was observed for AFW (**Figure 2C**), followed by 28 for ADW (**Figure 2D**).

Regarding leaf infection traits, the highest number of significant markers (34) was observed for NCL (**Figure 3A**), 8 significant markers were noted for NCNL (**Figure 3B**), and two significant markers distributed on chromosome 8 were found for NHL (**Figure 3C**),

### 3.4 Assigning significant markers to known genes

The results revealed that 27 SNP_S_ within 20 putative *M. truncatula* genes were associated with the recorded traits, as indicated in **Table 4**.

**Table 4:** Significant markers associated with seven morphological traits in alfalfa (*M. sativa*) accessions cultivated under the combination of salinity and *P. medicaginis* (Strain Pm8), identified using the *M. truncatula* genome as a reference.

Trait	Marker	Gene	Annotation
**NR**	S1.4_36648588	*MTR_1g082440*	Na+/H+ exchanger 1
**LMS**	S3.4_52698587	*MTR_3g112450*	Peptide transporter
**NHL**	S8.1_25315112	*MTR_8g469580*	Putative plant transposon protein domain-containing protein
**NHL**	S8.1_12523990	*MTR_8g032640*	Glycosyl hydrolase family 16 xyloglucan endotransglycosylase
**NCL**	S8.1_12523990	*MTR_8g032640*	Glycosyl hydrolase family 16 xyloglucan endotransglycosylase
**NCL**	S1.4_13820962	*MTR_1g037460*	Enhancer OF AG-4-like protein, putative
**NCL**	S2.4_8188471	*MTR_2g023280*	DUO pollen-like protein, putative
**NCL**	S4.2_56239116	*MTR_4g134340*	snRNA activating complex family protein
**NCL**	S8.1_2278244	*MTR_8g009590*	Fatty acid hydroxylase superfamily protein
**NCNL**	S2.4_32322717	*MTR_2g078010*	Ubiquitin-conjugating enzyme E2 35
**NCNL**	S4.2_23118171	*MTR_4g062300*	Hypothetical protein
**NCNL**	S8.1_33883534	*MTR_8g079365*	LRR and NB-ARC domain disease resistance protein
**AFW**	S1.4_22091088	*MTR_1g053065*	Transcription initiation factor IIB
**AFW**	S2.4_8188471	*MTR_2g023280*	DUO pollen-like protein, putative
**AFW**	S3.4_55239203	*MTR_3g118040*	Armadillo/beta-catenin-like repeat protein
**AFW**	S4.2_21507154	*MTR_4g058015*	Electron transporter, putative
**AFW**	S7.4_25562519	*MTR_7g069430*	Ribosomal RNA processing brix domain protein
**AFW**	S8.1_25579961	*MTR_8g470000*	LRR receptor-like kinase family protein
**ADW**	S1.4_22091088	*MTR_1g053065*	Transcription initiation factor IIB
**ADW**	S2.4_8188471	*MTR_2g023280*	DUO pollen-like protein, putative
**ADW**	S3.4_55239203	*MTR_3g118040*	Armadillo/beta-catenin-like repeat protein
**ADW**	S4.2_21507154	*MTR_4g058015*	Electron transporter, putative
**ADW**	S6.2_84793	*MTR_6g004040*	katanin p80 WD40 repeat subunit B1-like protein
**ADW**	S6.2_4127328	*MTR_6g013140*	Transmembrane protein, putative
**ADW**	S7.4_8552758	*MTR_7g025650*	F-box/RNI superfamily protein, putative
**ADW**	S7.4_25562519	*MTR_7g069430*	Ribosomal RNA processing brix domain protein
**ADW**	S8.1_44690997	*MTR_8g105880*	DUF241 domain protein

Length of medium stem (LMS, cm), number of ramification (NR), number of healthy leaves (NHL), number of chlorotic leaves (NCL), number of chlorotic necrotic leaves (NCNL), aerial fresh weight (AFW, g), and aerial dry weight (ADW, g).

Regarding the growth parameters, for the length of the main stem, one SNP on chromosome 3 (at locus 52698587) is linked to the homologous gene (*MTR_3g112450*), which encodes a peptide transporter. One gene (*MTR_1g082440*) is linked to one SNP located at locus 36648588 on chromosome 1, associated with number of ramifications, and encodes a Na^+^/H^+^ exchanger.

For the biomass parameters, five common genes were identified in both AFW and ADW traits, with the highest significant marker on chromosome 7 at locus 25562519 linked to the gene *MTR_7g069430*, which encodes the ribosomal RNA processing brix domain protein. Four other genes were identified on chromosomes 1, 2, 3, and 4, at positions 22091088, 8188481, 55239203, and 1507154, respectively. These genes include transcription initiation factor IIB (*MTR_1g053065*), DUO pollen-like protein putative (*MTR_2g023280*), armadillo/beta-catenin-like repeat protein (*MTR_3g118040*), and electron transporter putative (MTR_4g058015). The gene *MTR_8g470000* on chromosome 8 at position 25579961 was associated with AFW only. It encodes for the LRR receptor-like kinase family protein.

However, the ADW trait differed from the AFW trait for the presence of five more significant markers that are linked to four genes. Two of these genes are located on chromosome 6 (*MTR_6g004040* and *MTR_6g013140*), encoding a katanin p80 WD40 repeat subunit B1-like protein and a transmembrane protein, respectively. Additionally, we identified one SNP on chromosome 7 linked to the homologous gene *MTR_7g025650* (F-box/RNI superfamily protein, putative), and another SNP on chromosome 8 linked to the homologous gene *MTR_7g025650* (DUF241 domain protein).

With respect to infection parameters, the SNPs associated with the number of healthy leaves located at positions 25315112 and 12523990 on the same chromosome 2 were in the coding region of the putative plant transposon protein domain-containing protein (*MTR_8g469580*) and the glycosyl hydrolase family 16 xyloglucan endotransglycosylase linked to the homologous gene *MTR_8g032640*.

Five SNPs were significantly associated with the number of chlorotic leaves, distributed on chromosomes 1, 2, 4, and 8. One was the marker S1.4_13820962, which is close to the enhancer OF AG-4-like protein, putatively homologous to gene *MTR_1g037460*. The DUO pollen-like protein, putatively homologous to gene *MTR_2g023280*, was linked to the marker S2.4_8188471, and the snRNA activating complex family protein, homologous to gene *MTR_4g134340*, was linked to the marker S4.2_56239116. On chromosome 8, two markers (S8.1_12523990 and S8.1_2278244) were linked to glycosyl hydrolase family 16 xyloglucan endotransglycosylase and fatty acid hydroxylase superfamily protein, linked to homologous genes *MTR_8g032640* and *MTR_8g009590*, respectively.

For NCNL, we identified three genes. One of them is homologous to the gene encoding ubiquitin-conjugating enzyme E2 35, located on chromosome 2. Another is homologous to the gene encoding a hypothetical protein, located on chromosome 4 (*Medtr4g009410*). The third gene encodes an LRR and NB-ARC domain disease resistance protein, located on chromosome 8.

## 4 Discussion

Analyzing the morpho-physiological variation in alfalfa plants under stress conditions has been regarded as a reliable method for assessing its stress tolerance. This process represents a pivotal phase in the development of forthcoming breeding programs (Annicchiarico, 2006; Annicchiarico et al., 2022). Salinity and *Phoma medicaginis* infection are major issues for alfalfa that affect its growth and productivity. Previous studies have demonstrated that the alfalfa response to salinity varies greatly among accessions (Badri et al., 2021; Jabri et al., 2021).

This study represented an unprecedented example of exploring the morphological variation of alfalfa in response to the simultaneous presence of biotic and abiotic stressors. Our findings revealed a remarkable range of morpho-physiological variation within the examined collection of alfalfa genotypes when subjected to simultaneous stressors (salt and strain Pm8 infection) and, overall, the current results demonstrated significant genetic variation for most of the recorded traits. These findings are in accordance with the conclusions drawn by Annicchiarico et al. (2022) on the same population grown under drought stress. Badri et al. (2023) tested the effect of *P. medicaginis* on the variation in agronomic traits of different *Medicago* species, reporting that most of the variation in the measured parameters was explained by the infection treatment. Zaidi et al. (2021) demonstrated that the number of healthy leaves (recorded among the infection parameters of alfalfa genotypes) varied significantly among the genotypes infected by *P. medicaginis*, which is consistent with the current observations.

In this study, the highest heritability was found for NHL, suggesting that this trait is largely governed by genetic effects. Badri et al. (2023) found that most evaluated traits in infected *Medicago* species had high heritability values, suggesting their potential as useful criteria for identifying *P. medicaginis*-tolerant lines. Djaman et al. (2020) found a moderate heritability for the length of main stem in alfalfa, owing to the environmental effects affecting this character besides the genetic ones. The positive correlations observed among phenotypic traits in alfalfa accessions under combined stress of salinity and *P. medicaginis* infection suggest that these conditions influence multiple traits similarly, leading to correlated responses. The increase in chlorotic and chlorotic necrotic leaves may indicate an enhanced immune response to fungal infection, implying a complex interaction between abiotic and biotic stressors. This interaction may increase plant susceptibility to fungal infection or worsen the effects of saline stress on plant physiology. Additionally, the correlations between phenotypic traits may indicate adaptive strategies employed by plants to mitigate environmental stress damage, such as increasing chlorotic leaves.

In this study, GWAS allowed to identify 110 SNP markers associated with seven morphological traits of alfalfa grown under combined salinity and *P. medicaginis* infection. To our knowledge, this was the first attempt to locate genes regulating morpho-physiological traits in alfalfa under simultaneous abiotic (salinity) and biotic (strain Pm8 infection) stresses. Liu and Yu (2017) identified 42 SNPs significantly associated with salt tolerance using 198 alfalfa accessions with four physiological traits (dry weight, plant height, leaf chlorophyll content and stomatal conductance). Only 10 SNPs have been identified in a panel of 179 lines of alfalfa for *Verticillium* wilt tolerance (Yu et al., 2017). Several research studies used diploidized models and the reference genome of *M. truncatula* to identify putative genes in alfalfa under stress (Biazzi et al., 2017; Medina et al., 2020; Lin et al., 2021).

In this study, 24 putative genes have been linked to homologue molecular and biological responses to combined stresses. The GWAS for the length of the main stem showed a close association of this character with the gene *MTR_3g112450*, which encodes a peptide transporter necessary for the organic nitrogen (N) supplies and mediated N use efficiency under salt stress (Fang et al., 2017). Similarly, Wang et al. (2022) demonstrated the high expression and regulation of the peptide transporter family induced in alfalfa grown in salinity stress. Overall, the peptide transporter plays a pivotal role in our study to alleviate salt stress in alfalfa by facilitating the uptake of organic nitrogen compounds, which contribute to osmotic adjustment, antioxidant defense, nutrient uptake, and gene regulation (Chen et al., 2021). This coordinated response not only boosts the elongation of the main stems but also enhances the plant’s ability to withstand and recover from salt-induced damage (Wang et al., 2022).

Putative candidate genes that were linked to SNPs located in the first chromosome encode for the Na^+^/H^+^ exchanger, and were associated with the number of ramifications, which plays an important role for alfalfa salt tolerance (Guiza et al., 2022). The Na^+^/H^+^ exchanger, located in both the plasma and vacuolar membranes, plays a pivotal role in plants by actively expelling excess sodium ions (Na^+^) from the cytosol or sequestering them into the tonoplast. This mechanism is essential for mitigating the harmful effects of sodium toxicity (Yang et al., 2005). A study conducted by Li et al. (2011) showed the effective expression of SsNHX (Na^+^/H^+^ exchanger) in transgenic alfalfa, which could grow in high concentrations of NaCl (up to 400 mM). In addition, Lei et al. (2015) reported that alfalfa genotypes tolerant to salinity maintain moderately more stable expression levels of genes related to Na^+^/K^+^ transport and Na^+^/H^+^ exchanger. However, tonoplast and plasma membrane antiporters actively remove sodium ions from the cytosol. This process is powered by the proton-motive force, which is produced by the H^+^-ATPase in the plasma membrane, as well as the H^+^-ATPase and H^+^-pyrophosphatase in the vacuolar membrane. Notably, research has demonstrated that Na^+^/H^+^ exchanger was identified within the late endosome/vacuolar compartment, and it has been suggested that its functions may encompass sodium transport, control of water movement, regulation of vesicle volume, and possibly play a role in osmo-tolerance (Silva and Gerós, 2009). Our finding suggested that Na^+^/H^+^ exchanger gene holds significant importance in enhancing the proliferation of ramification in alfalfa under salt stress conditions. Its pivotal role lies in facilitating the exchange of nitrogen and hydrogen ions, crucial for mitigating the detrimental effects of salinity on plant growth (Li et al., 2018). This gene ensures the maintenance of optimal nutrient levels and pH balance within plant cells (Guiza et al., 2022), promoting the development of a greater number of ramifications in alfalfa even amidst challenging salt stress environments.

GWAS identified the presence of five common markers associated with both AFW and ADW. Among these markers, *MTR_1g053065* was linked to a homologous of the transcription initiation factor IIB, which was found to be involved in pollen and endosperm development in *Arabidopsis* (Dubos et al., 2010). Similarly, Xu et al. (2019) noted that the comparative transcriptome analysis of five alfalfa genotypes grown under cold stress revealed a gene encoding TFIIB (transcription factor IIB). This marker was differentially expressed in *Brassica napus* under drought stress using GWAS mapping (Khanzada et al., 2020). In addition, the putative electron transporter was significantly associated with ADW and AFW. *MTR_4g058015* is also involved in the regulation of Na^+^/K^+^ in alfalfa under salt stress (Li et al., 2010), and was also reported in salt tolerance of rice (Patishtan et al., 2018). Thus, the essential role of TFIIB in our study resulted in the regulation of ionic transport and osmotic adjustment leading to the enhancement of nutrient acquisition by alfalfa grown under salinity.

Furthermore, the association analysis for the ADW revealed that this trait was highly associated with the putative genes encoding for F-box/RNI superfamily protein on chromosome 7. Lechner et al. (2006) noted that F-box protein is a crucial element within the ubiquitin ligase complex, facilitating the ubiquitination process of specific target proteins. This molecular mechanism plays a pivotal role in regulating cellular processes associated with plant defense to fungi infection. This may explain the high significance of the marker encoding this gene in the current study, as it mediates the degradation of specific proteins involved in susceptibility pathways. Through targeted protein degradation, the F-box protein regulates key factors that contribute to the virulence of *P. medicaginis* or the susceptibility of alfalfa. Similarly, a candidate gene encoding F-box protein was identified in alfalfa, using GWAS, as a negative regulator of resistance to root rot caused by *Aphanomyces* spp (Bonhomme et al., 2014).

The plant *NBS_LRR* gene family contains a large class of disease resistance genes (Yu et al., 2020). In this study, a LRR receptor-like kinase family protein was positively associated with AFW and a LRR and NB-ARC domain disease resistance protein was linked to a marker located in chromosome 8 for NCNL. These genes encode proteins that are involved in plant disease resistance (Li et al., 2019). GWAS revealed multiple QTLs related to *Verticillium* wilt resistance on alfalfa chromosome 8 (Yu et al., 2017). These results imply that alfalfa resistance can be improved by stacking major R genes/QTLs for multiple pathogens associated with AFW trait (Fuchs, 2017).

The annotations of the homologue gene *MTR_4g062300* (S4.1_23118171) associated with NCNL pertain to hypothetical proteins with unknown functions. Earlier research has revealed that genes responsible for these hypothetical proteins were notably upregulated either at the transcript or translation level following prolonged exposure to high salt levels. This suggests that certain hypothetical proteins play a role in enhancing salt tolerance in forage plants, as demonstrated by Lin et al. (2021).

Five genes associated with the NCL trait were found to be located on *M. truncatula* chromosomes 1, 2, 4 and 8. Among them, one encodes the protein involved in DUO pollen-like protein. DUO acquired sperm lineage-specific expression in the common ancestor of land plants leading to sperm with distinct morphologies (Warman et al., 2020). The other one encodes for fatty acid hydroxylase superfamily protein, which has a metabolism control function (Marchler-Bauer et al., 2017). Preserving membrane integrity and fluidity is a crucial aspect of stress adaptation. This involves the collaboration between membrane components and the lipid composition to ensure the resilience of the membrane structure when facing challenging conditions. In this study, under combined stress, alfalfa cell membrane fluidity significantly decreased by the appearance of chlorosis spots on leaves, which is a symptom of salinity stress. As a result, the normal physiological function of membrane-bound proteins is altered (Liu et al., 2015). Previous studies reported the presence of the same putative gene using the GWAS approach in maize grown at low temperatures (Zhang et al., 2020), as well as in wheat under salt and drought stresses (Urbanavičiūtė et al., 2021).

A glycosyl hydrolase family 16 xyloglucan endotrans-glycosylase was found to be linked to the marker S8.1_12523990 associated to NHL. Recent studies have shown that xyloglucan endotrans-glycosylase, a prominent cell wall modifying enzyme, causes cell expansion, with loosening/reinforcing cell walls, particularly in response to environmental stress (Panahabadi et al., 2022). They demonstrated that glycosyl hydrolase family 16 xyloglucan endotrans-glycosylase was included in brassinolide hormone biosynthesis and pathways. Besides, Konkolewska et al. (2023) mentioned that the glycosyl hydrolase family 16 xyloglucans are intricate hemi-cellulosic polysaccharides that undergo biosynthesis within the Golgi apparatus before being transported to the cell wall of alfalfa. These molecules play essential roles in processes such as cell growth and expansion, energy metabolism, and signaling. Our finding suggested that the glycosyl hydrolase family 16 xyloglucan endotrans-glycosylase plays a crucial role in maintaining the number of healthy leaves in alfalfa during fungal infection by enhancing cell wall integrity, activating defense responses, regulating leaf morphology, and promoting nutrient transport.

## 5 Conclusions

Understanding stress tolerance mechanisms and genes in alfalfa helps identifying suitable genotypes for regions with high level of salinity and *P. medicaginis* infection, ensuring forage quality and quantity. Most traits assessed in the current study showed high heritability due to genetic factors, suggesting that the five alfalfa genotypes identified as tolerant to the combined stresses could be a valuable resource for the breeding. Using GWAS, we identified a total of 24 significant markers associated with seven traits. Notably, markers such as S8.1_12523990, S4.1_23118171, and S8.1_2278244 were linked to traits related to cell wall rigidity and cellular metabolism. We also found a marker associated with ionic transport. Some markers were linked to the *NBS_LRR* gene family, which plays a role in biomass and infection parameters, possibly contributing to alfalfa disease resistance.

The validation of the identified markers and genes through functional studies and biochemical assays, coupled with the exploration of gene overexpression techniques, represents a crucial step in marker-assisted selection (MAS). This process offers potential avenues for future research, such as investigating gene editing techniques to manipulate key genes associated with combined stress tolerance. Similarly, the most promising combined-tolerant alfalfa genotypes, containing numerous candidate genes for target traits, could be selected as parents in future crosses aimed at producing genetically superior alfalfa varieties with possibly multiple candidate genes for salinity tolerance, thereby allowing for sustained productivity even in saline-affected soils. Besides, resistance to *P. medicaginis* infection could mitigate crop losses and reduce the need for chemical interventions. However, ensuring the stability and adaptability of stress-tolerant traits across different environmental conditions and geographic regions will be essential for the widespread adoption of new alfalfa varieties

## Data availability statement

The original contributions presented in the study are included in the article/supplementary materials. Further inquiries can be directed to the corresponding author.

## Author contributions

WM: Data curation, Formal analysis, Software, Validation, Writing – original draft, Writing – review & editing. CJ: Conceptualization, Data curation, Methodology, Validation, Writing – review & editing. NJ: Data curation, Methodology, Validation, Writing – review & editing. NM: Methodology, Validation, Writing – review & editing. AG: Methodology, Validation, Writing – review & editing. NZ: Methodology, Validation, Writing – review & editing. GB: Methodology, Validation, Writing – review & editing. EK: Methodology, Validation, Writing – review & editing. ND: Resources, Validation, Writing – review & editing. LP: Resources, Validation, Writing – review & editing. MH: Conceptualization, Validation, Writing – review & editing. PA: Resources, Validation, Writing – review & editing. MS: Formal analysis, Software, Validation, Writing – review & editing. NL: Conceptualization, Funding acquisition, Validation, Writing – review & editing. MB: Conceptualization, Data curation, Funding acquisition, Investigation, Methodology, Project administration, Supervision, Validation, Writing – original draft, Writing – review & editing.

## References

[B501] Ahsan M. Z. Majidano M. S. Bhutto H. Soomro A. W. Panhwar F. H. Channa A. R.. (2015). Genetic variability, coefficient of variance, heritability and genetic advance of some Gossypium hirsutum L. accessions. JAS 7, 147. doi: 10.5539/jas.v7n2p147

[B1] Annicchiarico P. (2006). Diversity, genetic structure, distinctness and agronomic value of Italian lucerne (*Medicago sativa* L.) landraces. Euphytica 148, 269–282. doi: 10.1007/s10681-005-9024-0

[B2] Annicchiarico P. Barrett B. Brummer E. C. Julier B. Marshall A. H. (2015a). Achievements and challenges in improving temperate perennial forage legumes. Crit. Rev. Plant Sci. 34, 327–380. doi: 10.1080/07352689.2014.898462

[B5] Annicchiarico P. Nazzicari N. Bouizgaren A. Hayek T. Laouar M. Cornacchione M.. (2022). Alfalfa genomic selection for different stress-prone growing regions. Plant Genome 15, e20264. doi: 10.1002/tpg2.20264 36222346 PMC12807203

[B4] Annicchiarico P. Nazzicari N. Li X. Wei Y. Pecetti L. Brummer E. C. (2015b). Accuracy of genomic selection for alfalfa biomass yield in different reference populations. BMC Genom. 16, 1020. doi: 10.1186/s12864-015-2212-y PMC4667460 26626170

[B3] Annicchiarico P. Pecetti L. Abdelguerfi A. Bouizgaren A. Carroni A. M. Hayek T.. (2011). Adaptation of landrace and variety germplasm and selection strategies for lucerne in the Mediterranean basin. Field. Crops. Res. 120, 283–291. doi: 10.1016/j.fcr.2010.11.003

[B6] Arraouadi S. Badri M. Zitoun A. Huguet T. Aouani M. E. (2011). Analysis of NaCl stress response in Tunisian and reference lines of *Medicago truncatula* . Russ. J. Plant Physio. 58, 316–323. doi: 10.1134/S1021443711020026

[B7] Badri M. Ayadi A. Mahjoub A. Benltoufa A. Chaouachi M. Ranouch R.. (2023). Variability in responses to *phoma medicaginis* infection in a Tunisian collection of three annual *Medicago* species. Plant Pathol. J. 39, 171. doi: 10.5423/PPJ.OA.09.2022.0134 37019827 PMC10102567

[B8] Badri M. Rafik K. Jabri C. Ludidi N. (2021). Analysis of salinity tolerance in two varieties of *Medicago sativa* at the vegetative stage. J. Oasis Agric. Sustain. Dev. 3, 25–29. doi: 10.56027/JOASD.spiss042021

[B502] Benjamini Y. Hochberg Y. (1995). Controlling the false discovery rate: A practical and powerful approach to multiple testing. J. R. Stat. Soc Ser. B Methodol 57, 289–300. doi: 10.1111/j.2517-6161.1995.tb02031.x

[B9] Biazzi E. Nazzicari N. Pecetti L. Brummer E. C. Palmonari A. Tava A.. (2017). Genome-wide association mapping and genomic selection for alfalfa (*Medicago sativa*) forage quality traits. PloS One 12, e0169234. doi: 10.1371/journal.pone.0169234 28068350 PMC5222375

[B10] Bonhomme M. André O. Badis Y. Ronfort J. Burgarella C. Chantret N.. (2014). High-density genome-wide association mapping implicates an F-box encoding gene in M*edicago truncatula* resistance to *Aphanomyces euteiches* . New Phytol. 201, 1328–1342. doi: 10.1111/nph.12611 24283472

[B504] Bradbury P. J. Zhang Z. Kroon D. E. Casstevens T. M. Ramdoss Y. Buckler E. S. (2007). and TASSEL: software for association mapping of complex traits in diverse samples. Bioinformatics 23, 2633–2635. doi: 10.1093/bioinformatics/btm308 17586829

[B11] Castell-Miller C. V. Zeyen R. J. Samac D. A. (2007). Infection and development of *Phoma medicaginis* on moderately resistant and susceptible alfalfa genotypes. Can. J. Plant Pathol. 29, 290–298. doi: 10.1080/07060660709507472

[B12] Chen Z. Niu J. Guo Z. Sui X. Xu N. Kareem H. A.. (2021). Graphene enhances photosynthesis and the antioxidative defense system and alleviates salinity and alkalinity stresses in alfalfa (*Medicago sativa* L.) by regulating gene expression. Environ. Sci. Nano. 8, 2731–2748. doi: 10.1039/D1EN00257K

[B13] Djaman K. Owen C. Koudahe K. O’Neill M. (2020). Evaluation of different fall dormancy-rating alfalfa cultivars for forage yield in a semiarid environment. Agronomy 10, 146. doi: 10.3390/agronomy10010146

[B14] Djebali N. (2008). Etude des mécanismes de résistance de la plante modèle *Medicago truncatula* vis-à-vis de deux agents pathogènes majeurs des légumineuses cultivées : *Phoma medicaginis* et *Aphanomyces euteiches* . Université Toulouse III - Paul Sabatier, Castanet-Tolosan, France. 191.

[B15] Djébali N. Aribi S. Taamalli W. Arraouadi S. Aouani M. E. Badri M. (2013). Natural variation of *Medicago truncatula* resistance to *Aphanomyces euteiches* . Eur. J. Plant Pathol. 135, 831–843. doi: 10.1007/s10658-012-0127-x

[B16] Dubos C. Stracke R. Grotewold E. Weisshaar B. Martin C. Lepiniec L. (2010). MYB transcription factors in *Arabidopsis* . Trends Plant Sci. 15, 573–581. doi: 10.1016/j.tplants.2010.06.005 20674465

[B19] Fang Z. Bai G. Huang W. Wang Z. Wang X. Zhang M. (2017). The rice peptide transporter OsNPF7.3 is induced by organic nitrogen, and contributes to nitrogen allocation and grain yield. Front. Plant Sci. 8. doi: 10.3389/fpls.2017.01338 PMC5539172 28824674

[B505] Ferchichi Y. Sakhraoui A. Ltaeif H. B. Ben Mhara Y. Elimem M. Ben Naceur M.. (2021). Eco-geographical, morphological and molecular characterization of a collection of the perennial endemic species *Medicago tunetana* (Murb.) A.W. Hill (Fabaceae) from Tunisia. Plants 10, 1923. doi: 10.3390/plants10091923 34579454 PMC8468508

[B20] Filho C. C. F. Andrade M. H. M. L. Nunes J. A. R. Jarquin D. H. Rios E. F. (2023). Genomic prediction for complex traits across multiples harvests in alfalfa (*Medicago sativa* L.) is enhanced by enviromics. Plant Genome 16, e20306. doi: 10.1002/tpg2.20306 36815221 PMC12806953

[B21] Fuchs M. (2017). Pyramiding resistance-conferring gene sequences in crops. Curr. Opin. Virol. 26, 36–42. doi: 10.1016/j.coviro.2017.07.004 28755651

[B22] Guiza M. Benabdelrahim M. A. Brini F. Haddad M. Saibi W. (2022). Assessment of alfalfa (*Medicago sativa* L.) cultivars for salt tolerance based on yield, growth, physiological, and biochemical traits. J. Plant Growth Regul. 41, 3117–3126. doi: 10.1007/s00344-021-10499-9

[B23] Haddoudi I. Mhadhbi H. Gargouri M. Barhoumi F. Romdhane S. B. Mrabet M. (2021). Occurrence of fungal diseases in faba bean (*Vicia faba* L.) under salt and drought stress. Eur. J. Plant Pathol. 159, 385–398. doi: 10.1007/s10658-020-02169-5

[B24] Ibidhi R. Frija A. Jaouad M. Ben Salem H. (2018). Typology analysis of sheep production, feeding systems and farmers strategies for livestock watering in Tunisia. Small Rumin. Res. 160, 44–53. doi: 10.1016/j.smallrumres.2018.01.010

[B25] Jabri C. Zaidi N. Maiza N. Rafik K. Ludidi N. Badri M. (2021). Effects of salt stress on the germination of two contrasting *Medicago sativa* varieties. J. Oasis Agric. Sustain. Dev. 3, 13–18. doi: 10.56027/JOASD.spiss022021

[B26] Kaiwen G. Zisong X. Yuze H. Qi S. Yue W. Yanhui C.. (2020). Effects of salt concentration, pH, and their interaction on plant growth, nutrient uptake, and photochemistry of alfalfa (*Medicago sativa*) leaves. Plant Signal. Behav. 15, 1832373. doi: 10.1080/15592324.2020.1832373 33073686 PMC7671061

[B507] Kang Y. Sakiroglu M. Krom N. Stanton-Geddes J. Wang M. Lee Y.-C.. (2015). Genome-wide association of drought-related and biomass traits with HapMap SNPs in *Medicago truncatula* . Plant Cell Environ. 38, 1997–2011. doi: 10.1111/pce.12520 25707512

[B27] Khanzada H. Wassan G. M. He H. Mason A. S. Keerio A. A. Khanzada S.. (2020). Differentially evolved drought stress indices determine the genetic variation of *Brassica napus* at seedling traits by genome-wide association mapping. J. Adv. Res. 24, 447–461. doi: 10.1016/j.jare.2020.05.019 32577311 PMC7300156

[B28] Konkolewska A. Phang S. Conaghan P. Milbourne D. Lawlor A. Byrne S. (2023). Genomic prediction of seasonal forage yield in perennial ryegrass. Grassland Res. 2 (3), 841882. doi: 10.1002/glr2.12058

[B508] Larkin D. L. Lozada D. N. Mason R. E. (2019). Genomic selection—considerations for successful implementation in wheat breeding programs. Agronomy 9, 479. doi: 10.3390/agronomy9090479

[B29] Lechner E. Achard P. Vansiri A. Potuschak T. Genschik P. (2006). F-box proteins everywhere. Curr. Opin. Plant Biol. 9, 631–638. doi: 10.1016/j.pbi.2006.09.003 17005440

[B509] Lei L. Singh A. Bashline L. Li S. Yingling Y. G. Gu Y. (2015). Cellulose synthase interactive1 is required for fast recycling of cellulose synthase complexes to the plasma membrane in *Arabidopsis* . Plant Cell 27, 2926–2940. doi: 10.1105/tpc.15.00442 26443667 PMC4682321

[B513] Li X. Alarcón-Zúñiga B. Kang J. Nadeem Tahir M. H. Jiang Q. Wei Y.. (2015). Mapping fall dormancy and winter injury in tetraploid alfalfa. Crop Sci. 55, 1995–2011. doi: 10.2135/cropsci2014.12.0834

[B512] Li R. Shi F. Fukuda K. Yang Y. (2010). Effects of salt and alkali stresses on germination, growth, photosynthesis and ion accumulation in alfalfa (*Medicago sativa* L.). Soil Sci. Plant Nutr. 56, 725–733. doi: 10.1111/j.1747-0765.2010.00506.x

[B514] Li X. Wang G. Fu J. Li L. Jia G. Ren L.. (2018). QTL mapping in three connected populations reveals a set of consensus genomic regions for low temperature germination ability in *Zea mays* L. Front. Plant Sci. 9, 65. doi: 10.3389/fpls.2018.00065 29445387 PMC5797882

[B1000] Li T.-G. Wang B.-L. Yin C.-M. Zhang D.-D. Wang D. Song J.. (2019). The Gossypium hirsutum TIR-NBS-LRR gene GhDSC1 mediates resistance against *Verticillium* wilt. Mol. Plant Patho. 20, 857–876. doi: 10.1111/mpp.12797 PMC6637886 30957942

[B30] Li W. Wang D. Jin T. Chang Q. Yin D. Xu S.. (2011). The vacuolar Na^+^/H^+^ antiporter gene SsNHX1 from the halophyte salsola soda confers salt tolerance in transgenic alfalfa (*Medicago sativa* L.). Plant Mol. Biol. Rep. 29, 278–290. doi: 10.1007/s11105-010-0224-y

[B31] Lin S. Medina C. A. Norberg O. S. Combs D. Wang G. Shewmaker G.. (2021). Genome-Wide Association Studies identifying multiple loci associated with alfalfa forage quality. Front. Plant Sci. 12. doi: 10.3389/fpls.2021.648192 PMC8253570 34220880

[B32] Lin S. Medina C. A. Wang G. Combs D. Shewmaker G. Fransen S.. (2023). Identification of genetic loci associated with five agronomic traits in alfalfa using multi-environment trials. Theor. Appl. Genet. 136, 121. doi: 10.1007/s00122-023-04364-4 37119337

[B33] Liu C. Hao Z. Zhang D. Xie C. Li M. Zhang X.. (2015). Genetic properties of 240 maize inbred lines and identity-by-descent segments revealed by high-density SNP markers. Mol. Breed. 35, 146. doi: 10.1007/s11032-015-0344-z

[B35] Liu Z. Lan J. Li W. Ma H. (2023). Reseeding improved soil and plant characteristics of degraded alfalfa (*Medicago sativa*) grassland in loess hilly plateau region. China. Ecol. Eng. 190, 106933. doi: 10.1016/j.ecoleng.2023.106933

[B34] Liu X. P. Yu L. X. (2017). Genome-wide association mapping of loci associated with plant growth and forage production under salt stress in alfalfa (*Medicago sativa* L.). Front. Plant Sci. 8. doi: 10.3389/fpls.2017.00853 PMC5442208 28596776

[B36] Maiza N. Jabri C. Zaidi N. Rafik K. Khiari B. Djébali N.. (2021). High diversity of responses among *Medicago truncatula* lines to *Phoma medicaginis* infection. J. Oasis Agric. Sustain. Dev. 3, 52–57. doi: 10.56027/JOASD.spiss082021

[B37] Marchler-Bauer A. Bo Y. Han L. He J. Lanczycki C. J. Lu S.. (2017). CDD/SPARCLE: functional classification of proteins via subfamily domain architectures. Nucleic Acids Res. 45, 200–203. doi: 10.1093/nar/gkw1129 PMC5210587 27899674

[B515] Medina C. A. Hawkins C. Liu X.-P. Peel M. Yu L.-X. (2020). Genome-Wide Association and prediction of traits related to salt tolerance in autotetraploid alfalfa (*Medicago sativa* L.). Int. J. Mol. Sci. 21, 3361. doi: 10.3390/ijms21093361 32397526 PMC7247575

[B38] Panahabadi R. Ahmadikhah A. Farrokhi N. (2022). Genetic dissection of monosaccharides contents in rice whole grain using genome-wide association study. Plant Genome 6, e20292. doi: 10.1002/tpg2.20292 PMC12807434 36691363

[B39] Patishtan J. Hartley T. N. Fonseca de Carvalho R. Maathuis F. J. M. (2018). Genome-wide association studies to identify rice salt-tolerance markers. Plant Cell Environ. 41, 970–982. doi: 10.1111/pce.12975 28436093

[B516] Porter H. F. O’Reilly P. F. (2017). Multivariate simulation framework reveals performance of multi-trait GWAS methods. Sci. Rep. 7, 38837. doi: 10.1038/srep38837 28287610 PMC5347376

[B41] Sakiroglu M. Brummer E. C. (2017). Identification of loci controlling forage yield and nutritive value in diploid alfalfa using GBS-GWAS. Theor. Appl. Genet. 130, 261–268. doi: 10.1007/s00122-016-2782-3 27662844

[B40] Sakiroglu M. Sherman-Broyles S. Story A. Moore K. J. Doyle J. J. Charles Brummer E. (2012). Patterns of linkage disequilibrium and association mapping in diploid alfalfa (*M. sativa* L.). Theor. Appl. Genet. 125, 577–590. doi: 10.1007/s00122-012-1854-2 22476875 PMC3397135

[B517] Silva P. Gerós H. (2009). Regulation by salt of vacuolar H+-ATPase and H+-pyrophosphatase activities and Na+/H+ exchange. Plant Signal Behav. 4, 718–726. doi: 10.4161/psb.4.8.9236 19820346 PMC2801382

[B44] Thivierge M.-N. Bélanger G. Jégo G. Delmotte S. Rotz C. A. Charbonneau É. (2023). Perennial forages in cold-humid areas: Adaptation and resilience-building strategies toward climate change. Agron. J. 115, 1519–1542. doi: 10.1002/agj2.21354

[B45] Tlahig S. Karmous I. Mustapha G. Takwa J. Loumerem M. (2020). Effect of cutting time on the performance of alfalfa (*Medicago sativa* L.) genotypes cropped in arid environment. Pol. J. Environ. Stud. 30, 1817–1829. doi: 10.15244/pjoes/124757

[B46] Urbanavičiūtė I. Bonfiglioli L. Pagnotta M. A. (2021). One hundred candidate genes and their roles in drought and salt tolerance in wheat. Int. J. Mol. Sci. 22, 6378. doi: 10.3390/ijms22126378 34203629 PMC8232269

[B47] Wang Y. Yang P. Zhou Y. Hu T. Zhang P. Wu Y. (2022). A proteomic approach to understand the impact of nodulation on salinity stress response in alfalfa (*Medicago sativa* L.). Plant Biol. 24, 323–332. doi: 10.1111/plb.13369 34870352

[B48] Warman C. Panda K. Vejlupkova Z. Hokin S. Unger-Wallace E. Cole R. A.. (2020). High expression in maize pollen correlates with genetic contributions to pollen fitness as well as with coordinated transcription from neighboring transposable elements. PloS Genet. 16, e1008462. doi: 10.1371/journal.pgen.1008462 32236090 PMC7112179

[B49] Xu L. Tang X. Wang B. Xin X. Sun Q. Li Y.. (2019). Comparative transcriptome analysis of five Medicago varieties reveals the genetic signals underlying freezing tolerance. Crop Pasture Sci. 70, 273. doi: 10.1071/CP18165

[B50] Yang Q. Wu M. Wang P. Kang J. Zhou X. (2005). Cloning and expression analysis of a vacuolar Na^+^/H^+^ antiporter gene from Alfalfa. DNA Seq. 16, 352–357. doi: 10.1080/10425170500272742 16243725

[B518] Yu L.-X. Liu X. Boge W. Liu X.-P. (2016). Genome-Wide Association study identifies loci for salt tolerance during germination in autotetraploid alfalfa (*Medicago sativa* L.) using genotyping-by-sequencing. Front. Plant Sci. 7, 956. doi: 10.3389/fpls.2016.00956 27446182 PMC4923157

[B52] Yu L.-X. Zhang F. Culma C. M. Lin S. Niu Y. Zhang T.. (2020). Construction of high-density linkage maps and identification of quantitative trait loci associated with verticillium wilt resistance in autotetraploid alfalfa (*Medicago sativa* L.). Plant Dis. 104, 1439–1444. doi: 10.1094/PDIS-08-19-1718-RE 32150504

[B51] Yu L. X. Zheng P. Zhang T. Rodringuez J. Main D. (2017). Genotyping-by-sequencing-based genome-wide association studies on *Verticillium wilt* resistance in autotetraploid alfalfa (*Medicago sativa* L.). Mol. Plant Pathol. 18, 187–194. doi: 10.1111/mpp.12389 26933934 PMC6638244

[B53] Zaidi N. Jabri C. Maiza N. Rafik K. Khiari B. Djébali N.. (2021). Variation of *Medicago sativa* varieties tolerance to *Phoma medicaginis* infection.: *M. sativa* . J. Oasis Agric. Sustain. Dev. 3, 58–63. doi: 10.56027/JOASD.spiss092021

[B1001] Zhang T. Yu L. X. Zheng P. Li Y. Rivera M. Main D. Stephanie L. G.. (2015). Identification of loci associated with drought resistance traits in heterozygous autotetraploid alfalfa (*Medicago sativa* L.) using genome-wide association studies with genotyping by sequencing. PLoS ONE 10 (9), e0138931. doi: 10.1371/journal.pone.0138931 26406473 PMC4583413

[B54] Zhang H. Zhang J. Xu Q. Wang D. Di H. Huang J.. (2020). Identification of candidate tolerance genes to low-temperature during maize germination by GWAS and RNA-seq approaches. BMC Plant Biol. 20, 333. doi: 10.1186/s12870-020-02543-9 32664856 PMC7362524

